# Dental Litigation, Past and Present

**Published:** 1892-07

**Authors:** Joseph Head


### DENTAL LITIGATION, PAST AND PRESENT.[1]

BY JOSEPH HEAD, M.D., D.D.S.

THIS paper is a plea for absolute confidence in the Dental Protective Association. It will endeavor to show why every dentist should not only be a member, but an active partisan, working in every way to lighten the burden of those few men who by arduous devotion are saving each dentist an annual tax of at least a hundred dollars.

The Sheffield Tooth-Crown Company is operated by the same men who have in times past made the Dental Vulcanite Company so formidable.

Many dentists have thought to escape molestation by refraining from the manufacture of Richmond crown- and bridge-work, but they have not appreciated the danger which threatened, and which undoubtedly would have overwhelmed them, had not a few unselfish men, led by Dr. Crouse, championed the rights of all dentists at the risk of imprisonment and heavy fines.

The Tooth-Crown Company have patents not only on the Richmond crown- and bridge-work, but in addition, their patents, numbering almost a hundred, include innumerable well-known daily operations.

If these claims be sustained in court, no man can legally invest a tooth for soldering, or cement in any crown whatsoever, whether it be held by band, pin, or screw. Neither can he take impressions or articulations. Numerous other cases could be quoted, but these are sufficient to show the injustice and weakness of their claims.

But because they are unjust or illegal they are none the less to be feared. They can only be rendered harmless by vigorous opposition and close organization.

A certain Mr. Josiah Bacon, treasurer of the Goodyear Dental Vulcanite Company, is quoted to have said, "I don't care whether a patent is good or not, give me money enough and I will put it through." And he proved his assertion.

In 1852, Dr. Cummings tried to get a patent on the process of making rubber dentures. He was not the inventor, but picked up the idea from dentists with whom he associated. In fact, so little did he know of the process, that he included gutta-percha among the vulcanizable substances. And at one time he actually hired a

---

[1] Read before the Odontological Society of Pennsylvania, May 12, 1892.

mechanical dentist to teach one of his students to make rubber plates, he himself not being competent.

From 1852 to 1854 he tried to get the patent, but, not having sufficient influence, failed. Then he abandoned all attempts until 1858, when he tried again, and was again refused. Still he persisted, until, with the aid of Josiah Bacon, treasurer of the Goodyear Dental Vulcanite Company, in 1864, he succeeded in getting a patent, embodying not only his original claim, but all improvements made by the profession up to that time.

The patent was issued in absolute violation of law and justice.

Morally, he should not have had it, as he was not the real inventor. Technically, he should not have had it, because the laws forbid a patent to be issued for a method where a well-known substance is applied to a well-known process. This law directly applied to the case in point, as teeth had previously been flasked for the purpose of making plates of metal, which was poured into the mould. Again, the laws say, "No patent can be granted for a process that has been in general use two years previous to the petition." There are many other laws which applied, but these two alone were sufficient to render the patent unsound.

Mr. Bacon, who had bought this patent, did not care whether it was sound or not. He was about to prove his boast that a good or bad patent could be made successful with plenty of money. He wanted this, because it would extend the Goodyear patent nine years, so far as it related to artificial dentures.

When Cummings's patent was issued, there was a terrific storm of indignation throughout the country. Protests from individual dentists were presented on all sides, but there was no organization for vigorous opposition, as the Goodyear patent, covering the same ground, had yet several years to run. So the storm subsided, and the wily Vulcanite Company succeeded.

The story runs as follows: A certain Dr. Newbrough invented a process for hardening rubber by iodine, and issued licenses to several dentists offering to protect them from any suit that might be brought against them by Bacon & Co. Among these licensees was E. B. Gardner, against whom the Vulcanite Company brought suit for using iodized rubber, claiming that it was an infringement on the Cummings patent.

According to his agreement, Dr. Newbrough undertook Gardner's defence. Then Bacon proved his boast: he compromised with Newbrough, contracting to give him a share in the profits of the Vulcanite Company in exchange for all his patents. Next he engaged Newbrough's attorney to continue the Gardner case and finally permit the Vulcanite Company to win. Thus would the validity of the Cummings patent be established.

In proof of these statements it may be of interest to quote Dr. Newbrough's sworn testimony before the United States Supreme Court, bearing the date 1871, which may be found printed in full on page 190 of the *Dental Cosmos*, vol. xv. After a description of his patent he says, "Among my licensees was Dr. Benzoni E. Gardner, of Providence, R. I. In September, 1868, Dr. Gardner sent me a writ which had been served upon him at the suit of the Goodyear Dental Vulcanite Company and Josiah Bacon.

"Having been advised that my patents and their use involved no infringement of the Cummings patent, I was not unprepared for litigation, and promptly assumed the defence of my licensee, Dr. Gardner. The answer was filed after a preliminary injunction had been granted after argument. This ended the proceedings in the Gardner case, except as hereinafter explained. Previous to the Gardner suit in Rhode Island the same complainants had sued me in this district, and I was proceeding duly with my defence.

"No testimony was ever taken in the Gardner case except the affidavits used on motion for the injunction, but the testimony that afterwards went to make up the Gardner record was stipulated into it out of other suits. On the first day of July, A.D. 1869, a written compromise was executed between the Goodyear Dental Vulcanite Company, Josiah Bacon, and John H. Cheever, Henry E. Ficket, and myself, dismissing all the suits against myself, and I introduced into that compromise the dismissal of the suit against Gardner also, because, as I was the real defendant of his case, I regarded that as involving myself, and so settled it in the above-mentioned compromise by giving him a free license during the term of all the Goodyear Dental Vulcanite Company's patents. With the execution of this in writing all the then pending questions between the company, my licensees, and myself were finally settled and terminated, and from that time forward I have not in any manner controlled, directed, or participated in any defence or proceedings in the Gardner or the other suits above mentioned; but, on the contrary, my only interest in any of the matters has been that which I had in common with the company under the aforesaid compromise. After this compromise was effected, and as a matter of personal friendship to John A. Foster, and also to prevent the dentists from employing him against our then common interest, I did then get the company to take Mr. Foster as one of their lawyers, and he told me from time to time that the Goodyear Dental Vulcanite Company has paid him since July 1, 1869, for all services performed by him in the Gardner case; that after the argument of that case in Washington, before Judge Clifford, Mr. Foster told me that the company had paid him two thousand two hundred dollars for compiling the case. I remember this distinctly, because Mr. Foster told me at the same time that he would never have got his money from the company if he had not threatened 'to blow on the whole thing;' meaning, as I understood, to let the dentists know that it was a put-up job.

"I remember that shortly after my compromise the lawyers who were conducting the case concluded to put in all the testimony for both sides, so that, should the collusive suit be discovered, the testimony would be so fairly put in that their complicity in the matter would not involve themselves; and further, that after the case was ready for argument the dentists were to be informed of the fact, and induced to accept the case, as then prepared, so that they might take part or not as they chose.

(Signed) "J. B. Newbrough."

This contract was carried out September 8, 1870. No defence was made. The evidence was all in favor of the Goodyear Rubber Company, and from that time a new reign of terror was inaugurated. The Cummings patent had been legalized. Still they were not satisfied; but, to make their position more secure, Newbrough appealed the Gardner case to the Supreme Court. Bacon won this suit also. And thus the illegal Cummings patent would have been secure and impregnable if the contracting parties had not quarrelled.

Then the dentists combined and asked for a new trial. In view of the collusion exposed, the courts granted their request. That, however, the Vulcanite Company eventually won is a matter of history.

The dentists, with their loose and imperfect organization, were an easy prey. Henceforth each dentist who made rubber plates had to pay a tax varying from seventy-five to one hundred and twenty-five dollars a year, and was compelled to keep his books always open to the scrutiny of the Vulcanite agents. Failing to do this, he was liable to fine and imprisonment.

This story should teach an impressive lesson. Since unscrupulous corporations by mere force of money and influence can prey upon the individual dentist, can ruin the practitioner by expensive lawsuits, no matter how just his cause may be, since these facts are indisputable, there can be but one remedy. The dentists must also form a corporation for the purposes of self-defence. And this is what has been done. If for the use of one patent the old Vulcanite Company charged seventy-five to one hundred and twenty-five dollars per annum, what must necessarily be the result if the Tooth-Crown Company succeed in levying taxes on their eighty or ninety patents?

This company, up to this time, has been restrained by Dr. Crouse and his associates, who have had not only the courage to defy these powerful foes, but also the keenness to expose their methods. The Dental Protective Association lawyers are not to be bribed or corrupted, and thus suit after suit of the Tooth-Crown Company has been withdrawn in the face of overwhelming contrary evidence. They stand fully under the control of the Dental Protective Association.

Bacon would no doubt now be a moving factor in the new company if he had not been shot by a dentist whom he had hounded to the verge of desperation.

What, then, does this state of affairs mean? It means that the leaders of the Dental Protective Association have proved themselves worthy of implicit confidence. It means that any dentist now refusing to join is like the man who built his house upon the sands. For in a short time, when a full list of members of the association shall be published, those without the association will be left at the mercy of the Tooth-Crown agents, and will only be allowed to enter the protecting fold by paying an entrance fee of many times the present assessment.

The dentists are not asked to subscribe money towards fighting a doubtful battle: the victory has already been won. They are simply asked to pay ten dollars towards a campaign which in times past has saved them hundreds of dollars, and which in times to come will be a strong guarantee against oppression.

If, however, through selfishness or shortsightedness, many stay outside, they must not complain if they meet the fate of stragglers on the field of battle, who are captured or slain by the flying foe.

The Dental Protective Association is an assured success. And every dentist should gladly give ten dollars to an organization so capable and trustworthy.

